# Editorial: Minimally invasive cardiac surgery: state of the art and current challenges

**DOI:** 10.3389/fcvm.2023.1286868

**Published:** 2023-09-27

**Authors:** Tomas Holubec, Gry Dahle, Nikolaos Bonaros

**Affiliations:** ^1^ Department of Cardiovascular Surgery, Goethe University and University Hospital Frankfurt, Frankfurt/Main, Germany; ^2^ Department of Cardiothoracic Surgery, Oslo University Hospital, Oslo, Norway; ^3^ Department of Cardiac Surgery, Medical University of Innsbruck, Innsbruck, Austria

**Keywords:** minimally invasive cardiac surgery (MICS), coronary revascularization (MIDCAB and OPCAB), hybrid coronary revascularization, valve surgery, aortic surgery, endoscopic and robotic cardiac surgery

**Editorial on the Research Topic** Minimally invasive cardiac surgery: State of the art and current challenges

Minimally invasive cardiac surgery (MICS) has undergone a rapid evolution over the past three decades due to the significant progress in the development of emerging technologies and improved surgical techniques in the cardiovascular field.

Only about one-third of all cardiac surgery procedures are currently performed via small skin incisions (minithoracotomy and ministernotomy). However, the invasiveness of any cardiosurgical procedure cannot only be defined by access (skin incision) but also by the use of cardiopulmonary bypass with potential cardiac arrest and heart valve repair or valve-sparing operation (1). This positive trend continues to evolve, notably with the development of increasingly efficient endoscopic, robotic, and transcatheter procedures (2–4).

Driven by reduced surgical trauma, blood loss, pain, and hospital stay, as well as better cosmesis and quality of life, the considerable attention gained through the application of MICS is attributable to improved postoperative outcomes (5–8). However, some concerns remain with the technical challenges and the consequent prolonged intraoperative durations and risks of vascular complications, including thromboembolism, as well as associated neurological complications (9).

With this Research Topic, we aim to provide readers, clinicians, researchers, and developers a broad scientific and technological overview of the progress made with the various innovative minimally invasive surgical, reconstructive, and interventional approaches to coronary arteries, heart valves, and aortas, since their introduction about 30 years ago.

An excellent didactic summary of 10 years of experience with MICS, especially endoscopic, incorporating seven lessons learned is provided by Ahmad et al. Based on their broad experience, the authors suggest MICS can be safely, effectively, and reproducibly performed by a wide range of surgeons. Additionally, it can serve as a good template for establishing MICS and accelerating the learning curve while improving patient outcomes. From the same two institutions, an interesting overview about the experience with minimally invasive direct coronary artery bypass grafting (MIDCAB) is published by Monsefi et al.. The authors present the short-term results of 234 patients undergoing MIDCAB between 2017 and 2021 with a 30-day mortality of 1.7%. This study confirms the aforementioned fact that the recently started MICS programme can offer very good outcomes to patients. These short-term results are even comparable with the largest ever published MIDCAB cohort (10).

The gold standard treatment of primary degenerative mitral valve insufficiency is surgical valve repair, which nowadays is performed predominantly in MICS and increasingly in three-dimensional endoscopic fashion (2). Elderly patients suffering from additional atherosclerosis bear an increased risk due to retrograde arterial perfusion. Selective cannulation of the right axillary artery and herewith antegrade perfusion may be of benefit. Petersen et al. performed a study comparing short-term outcomes of this perfusion strategy with standard retrograde femoral perfusion. They conclude that patients with a higher perioperative risk and severe atherosclerosis would benefit from antegrade axillary perfusion.

Since its introduction in 1992, aortic valve (AV) reimplantation (David procedure) has become the standard technique for patients suffering from aortic root aneurysm with or without AV insufficiency and has produced excellent short- and long-term results (11). A quarter century experience with this valve-sparing operation from a teaching centre is reported by Sromicki et al. The 30-day mortality of their cohort of 131 patients was 2%. Freedom from reoperation at 5 and 10 years was 93.5% ± 2.4% and 87.0% ± 3.5%, respectively. These results are comparable with other mid-volume centres (12); however, they are not as exceptional as the results from the Toronto group. In our opinion, the explanation for these exceptional and almost unreproducible results is the extreme selection of patients over the increasing course of time.

Adding a minimal access to the aortic valve-sparing, this procedure can be then considered as a great representative of MICS and is of major benefit to the patients. Shrestha et al. compared patients undergoing elective isolated David procedure via ministernotomy (42 patients) with full sternotomy (220 patients). Despite the fact that perioperative outcomes (cardio-pulmonary bypass and aortic cross-clamp time) were statistically relevantly shorter in the full sternotomy group, no difference was found in short- and long-term postoperative outcomes, including valve performance.

The MICS has not yet been adopted in aortic arch repair and even less in the surgery of acute type A aortic dissection (13). Since January 2019, Xie et al. have operated all obese (BMI ≥30 kg/m^2^) patients with acute type A aortic dissection using a self-made triple-branched stent-graft for total arch replacement via partial upper sternotomy. In their study, 35 patients underwent full sternotomy, and 30 partial upper sternotomy. The latter strategy was proved to be safe, effective, and superior to full sternotomy in terms of blood loss, postoperative blood transfusion, and respiratory complications.

The Research Topic was rounded off by three interesting case reports. Pojar et al. present a remarkable case of successful robotic repair of unroofed coronary sinus, which was accomplished using an excellent high-resolution video. Salamate et al. publish an extraordinary technically challenging case of video-assisted minimally invasive mitral and pulmonary valve replacement as a reoperation in a patient with situs invs. totalis. This case report was also accomplished using an excellent high-resolution video. Finally, Wu et al. present a remarkable case of successful minimally invasive bicuspid AV repair through right-anterior minithoracotomy.

The aim of this Research Topic was to assess current progress in MICS of the coronary arteries, heart valves, and aorta. Nine papers were accepted and collected in this Research Topic, and to date, have been seen by over 7,500 readers. These publications confirm the steady progress of this approach and demonstrate that MICS is safe and feasible. However, MICS is still relatively uncommon, being confined mainly to specialist centres. In our opinion, MICS is the approach of the future and is *a priori* suitable for every patient and every pathology; nevertheless, precise selection and rigorous preoperative planning are essential. More in-depth analyses on larger groups are also required.
